# NRF2 Regulates HER2 and HER3 Signaling Pathway to Modulate Sensitivity to Targeted Immunotherapies

**DOI:** 10.1155/2016/4148791

**Published:** 2015-12-07

**Authors:** Hilal S. Khalil, Simon P. Langdon, Ibrahim H. Kankia, James Bown, Yusuf Y. Deeni

**Affiliations:** ^1^SIMBIOS, School of Science, Engineering and Technology, Abertay University, Dundee DD1 1HG, UK; ^2^Pathology Division, Institute of Genetics and Molecular Medicine, University of Edinburgh, Edinburgh EH4 2XU, UK

## Abstract

NF-E2 related factor-2 (NRF2) is an essential transcription factor for multiple genes encoding antioxidants and detoxification enzymes. NRF2 is implicated in promoting cancer therapeutic resistance by its detoxification function and crosstalk with proproliferative pathways. However, the exact mechanism of this intricate connectivity between NRF2 and growth factor induced proliferative pathway remains elusive. Here, we have demonstrated that pharmacological activation of NRF2 by *tert*-butylhydroquinone (tBHQ) upregulates the HER family receptors, HER2 and HER3 expression, elevates pAKT levels, and enhances the proliferation of ovarian cancer cells. Preactivation of NRF2 also attenuates the combined growth inhibitory effects of HER2 targeting monoclonal antibodies, Pertuzumab and Trastuzumab. Further, tBHQ caused transcriptional induction of HER2 and HER3, while SiRNA-mediated knockdown of NRF2 prevented this and further caused transcriptional repression and enhanced cytotoxicity of the HER2 inhibitors. Hence, NRF2 regulates both HER2 and HER3 receptors to influence cellular responses to HER2 targeting monoclonal antibodies. This deciphered crosstalk mechanism reinforces the role of NRF2 in drug resistance and as a relevant anticancer target.

## 1. Introduction

The receptor tyrosine kinases (RTKs) are key drivers of normal cellular proliferation, differentiation, and survival, as well as determinants of cancer initiation, maintenance, and progression [1–4]. Dimerization and stimulation of the intrinsic tyrosine kinase in RTKs lead to the phosphorylation of tyrosine residues in the intracellular domain of the receptors. The phosphotyrosine residues serve as docking sites to recruit a number of signal adapter proteins containing the so-called SH2 and PTB domains, which link RTKs to different cellular signaling pathways such as PI3K/AKT/mTOR, MAPK, and STAT pathways [5, 6]. Among the RTK superfamily receptors are the type I RTKs that belong to the epidermal growth factor receptor (EGFR or HER) family. The HER receptor network contains four members (HER1, HER2, HER3, and HER4) whose activation kinetics depend significantly on their expression levels which vary across different cells and cancers [7]. Likewise it is these variations combined with receptor interaction that drive and confer complexity in the HER receptor family behaviour.

The two receptors, HER2 and HER3, are nonautonomous and possess certain defining features, in that HER2 has autokinase activity but no known ligands, and HER3 is a pseudokinase receptor that lacks tyrosine kinase activity. These features define the interaction between the HER2 and HER3 receptors and for forming active homodimer and heterodimer complexes. Specifically, mutation or increased gene copy number leads to overexpression of HER2 receptors in cancer cells causing constitutive activation of proliferative pathways in the absence of ligand through homodimerization and RTK autophosphorylation [1, 5, 6, 10]. HER2 functions as the shared coreceptor for EGFR, HER3, and HER4 receptors, and these heterodimeric complexes are activated by the partner ligands [11, 12]. Moreover, HER2 is the preferred heterodimerization partner of HER3, whilst HER2 overexpression is believed to enhance the signaling from HER3 receptor in response to binding of its specific ligand, neuregulin. As such, HER2-HER3 heterodimers are known to be the strongest elicitors of the PI3K/AKT/mTOR pathway [13–17]. Coexpression of the different receptors, the diversity in their ligand-independent and ligand-dependent activation, variation in their preference towards dimerization partners, and receptor-dependent specificity in cells play a major role in both redundancy in the HER network interaction and effective drug target identification [17–21]. Further complexity in HER2/HER3 activation and signaling arises from the complex transcriptional and posttranslational coregulation of HER2/HER3 receptors and their ligands following HER receptor specific targeted therapies which often lead to inconsistent tumour responses [13, 17, 21].

Nuclear factor- (erythroid-derived 2-) like 2 (NRF2) is a leucine zipper transcription factor and the master regulator of the antioxidant response (AR) pathway. It drives both basal and oxidative stress-induced transcription of a battery of phases I, II, and III detoxification enzymes and cytoprotective genes [22–24] as well as other genes of the metabolic and signal transduction pathways [14, 23, 25]. This is achieved by heterodimerization of NRF2 with small MAF proteins and binding to some genome* cis*-acting factors called antioxidant response elements (ARE) or electrophile response elements (EpREs) within the promoters of its target genes [26, 27]. Under basal conditions, only a low level of free NRF2 is available in the cytoplasm with some translocating into the nucleus to drive the basal transcription of target genes.

Like the HER receptors [28–31], NRF2 is a recognised agent in cellular proliferation and adaptation to reactive oxygen species (ROS) and in conferring therapeutic resistance to cancers [32–34]. Importantly, NRF2 activation and KEAP1 inactivation mutations leading to permanent constitutive adaptive activation of the NRF2 pathway are frequently observed in cancers [35–37]. Also several therapeutic strategies such as anticancer radio- and chemotherapy greatly depend on ROS manipulation to induce cytotoxicity. Paradoxically, there is a growing body of evidence implicating HER2/HER3, NRF2, and ROS in the promotion of cellular proliferation and therapeutic resistance in cancer cells [38, 39]. Cancer cells have been shown to evolve intricate mechanisms of cellular resistance towards both ROS and other cellular damaging agents as demonstrated by a very robust antioxidant sensing and ROS neutralising mechanisms as well as a highly efficient cytoprotective systems [33, 34, 40–42].

ROS is long not only recognised as the regulator of NRF2 stability and activity but has also been shown to trigger both the AR and the HER family receptor pathways with concomitant transcriptional upregulation of HER2/HER3 complexes and subsequent activation of their functions [30, 31, 43, 44]. Hence, ROS might serve as the point of convergence and as such establish cross relationship between the two pathways. Furthermore, components of the receptor regulated PI3K and MAPK have been shown to regulate NRF2 function [45–47], while many aspects of RTK signaling are regulated by ROS whose levels are directly modulated by NRF2 function [48, 49].

Since NRF2 is a transcription factor to several hundreds of genes, including proto-oncogenes, it is feasible that HER2/HER3 receptors are transcriptional targets of NRF2 via direct or indirect means involving ROS. Thus this study aims to investigate this and identify crosstalk between the NRF2 dependent AR pathway and the HER2/HER3 receptors signaling pathway, in order to determine their potential interdependence in eliciting cellular proliferation, cytoprotection, and responses to therapies.

By generating gene transcriptional reporter assays, carrying out pharmacological activation or SiRNA knockdown of NRF2, and performing HER2/HER3 functional inhibition and activation strategies, we have identified a direct node of functional integration of the two pathways in our ovarian cancer cell model which converges at NRF2. We demonstrated that inhibition of NRF2 leads to disruption of the antioxidant pathway and attenuation of HER2/HER3 signaling and that this is as a consequence of transcriptional repression of both* HER2* and* HER3* genes. Furthermore, we have demonstrated that this functional link could be utilised to either sensitise or reproduce resistant responses in our cell model. Thus, this study reveals a new mechanism of crosstalk between AR and HER2/HER3 pathways and opens up novel avenues of targeting and manipulating the NRF2-AR to uncouple and sensitise HER2/HER3 pathways resistant ovarian cancer cells to targeted immunotherapeutics.

## 2. Materials and Methods

### 2.1. Cell Lines, Culture Conditions, and Treatments

Human ovarian cancer cell lines PEO1 and SKOV3 were maintained in RPMI 1640 media (Gibco Invitrogen) supplemented with 10% foetal bovine serum (FBS), 2 mM glutamine, 1 mM sodium pyruvate, 100 *μ*g/mL streptomycin, and 100 U/mL penicillin in an atmosphere of 5% CO_2_ and incubated at 37°C. Before experimental treatments, cells were grown for 24 h in RPMI 1640 media prepared as above but replacing FBS with 5% double charcoal stripped FBS (Fisher). Heregulin-*β*1 (HRG, Sigma) was used by preparing 1 *μ*mol/L stock solution made with 5% trehalose and 10% FBS in phosphate buffered saline (PBS) and diluted to a final concentration of 1 nmol/L with media during treatments. Monoclonal antibodies targeting HER2 receptor, Pertuzumab and Trastuzumab (RTKi), were used by directly diluting the drugs in media to a final concentration of 20 *μ*g/mL. Tert-butylhydroquinone (tBHQ) stock solution (Sigma) was made with Dimethylsulfoxide (Fisher) and diluted to a final concentration as required with media. For ROS detection, 2′,7′-Dichlorofluorescin diacetate (DCFDA, Sigma) solution was prepared with Dimethylsulfoxide in amber tubes to a concentration of 50 mM and stored at −20°C in the dark until used. For cytotoxicity assay, 3-(4,5-Dimethylthiazol-2-yl)-2,5-Diphenyltetrazolium Bromide (MTT) was needed by making a stock solution of 5 mg/mL in PBS and filter sterilising it. The solution was stored at 4°C in the dark until used.

### 2.2. Reactive Oxygen Species (ROS) Detection

ROS detection assay was performed by using 2′,7′-Dichlorofluorescin diacetate (DCFDA) staining (Sigma). Briefly, cells were seeded in triplicate at a density of 0.2 × 10^5^ cells/well of opaque flat bottom 96-well tissue culture plates in 100 *μ*L media without phenol red and allowed to grow for 18 h. Following transfection, cells were washed with PBS and maintained in 100 *μ*L of phenol red-free medium and further incubated for 24 h. A 50 mM stock solution of DCFDA was added to each well containing 100 *μ*L preexisting media to achieve a final concentration of 25 *μ*M and incubated for 45 min at 37°C. Fluorescence signal intensities indicating ROS levels were recorded by taking readings using 96-well fluorescent multiplate reader (MODULUS, Promega) using excitation and emission spectra of 485 nm/535 nm. To normalise the fluorescence signal, cells in the same wells were stained with Coomassie brilliant blue stain (Sigma) for 1 h and washed with distilled water and 10% sodium dodecyl sulphate (SDS) solution was added to release the absorbed dye for 10 min while shaking. The absorbance values at 595 nm were then recorded using a multiplate absorbance reader (MODULUS, Promega) data used after normalising the fluorescence values.

### 2.3. Cloning and Expression Vectors Used in the Study

Closely 1.5 kb proximal promoter regions of HER2 and HER3 were cloned and used in the current study. The HER2 primer sequences used for each construct were HER2 forward: 5′-GTGCTCGAGGCAAGAAGGGTGCATTTTGAAG-3′ and HER2 reverse: 5′-GTCAAGCTTGTCTCTTGGATGGGCCATC-3′. The HER3 primer sequences used for each construct were HER3 forward: 5′-GTGCTCGAGGCCCTCTAGGTTGCATATCAATAGG-3′ and for HER3 reverse: 5′-GTCAAGCTTGAAAAGCAAGCCCAGCAC-3′. For cloning HER2 and HER3 promoters (prHER2 and prHER3, resp.), total genomic DNA was isolated from human cells using DNeasy Blood and Tissue Kit (Qiagen) and quantified using AstraGene microvolume spectrophotometer (AstraNet). 100 ng of the genomic DNA was used to amplify the promoter sequences (MyFi mix, Bioline) using relevant primers that incorporated* XhoI* and* HindIII* restriction endonuclease sites 5′ and 3′ ends of the amplified promoters, respectively. PCR conditions for promoter amplification were initial denaturation of 95°C for 7 min followed by 35 cycles of 95°C for 30 s for denaturation, 50°C for 30 s for annealing, and 72°C for 90 s for extension and a final extension for 10 min at 72°C. The PCR products were run and extracted from agarose gel (Qiagen), digested using* XhoI* and* HindIII* restriction enzymes (Promega), and ligated into PGL3 vector (Promega) to created HER2 and HER2 promoter constructs (prHER2 and prHER3, resp.) driving the expression of luciferase gene for utilisation in dual luciferase reporter assay (Promega). The integrity of cloned sequences was determined by sequencing the plasmids using commercial sequencing service (http://www.dnaseq.co.uk/). All cloned constructs were transfected into relevant cell lines using Lipofectamine 3000 (Life Technologies).

### 2.4. Protein Extraction and Immunoblotting

For immunoblotting, cells were seeded in 60 mm tissue culture plates and grown until being 70% confluent. At the time of protein harvest, cells were trypsinized (Gibco Invitrogen) and washed with PBS. Protein lysates were prepared using radio immune precipitation assay buffer (Pierce Biotech) supplemented with protease and phosphatase inhibitor cocktail (Pierce Biotech) and subjected to sonication of 2 cycles for 10 s at 50% pulse. The final mixture was shaken gently on ice for 15 min and the protein supernatant was obtained following centrifugation of the lysates at 14000 g for 15 min. Proteins obtained were quantified by Bradford assay (Sigma-Aldrich) using bovine serum albumin as a standard and sample buffer (Nupage LDS, Invitrogen) was added to protein lysates, heated at 70°C for 20 min, and stored at −20°C until further use. Once the protein lysates were prepared, they were loaded into wells of 4–12% gradient SDS-polyacrylamide gels (Nupage Bis-Tris gels, Life Technologies) and subjected to electrophoresis at 200 V for 1-2 h. Following this, proteins were transferred to polyvinylidene difluoride membranes (PVDF, GE Amersham) using the XCell SureLock Mini-Cell system (Invitrogen) at 50 V for 90 min and processed using a commercially available kit (WesternBreeze Chromogenic Immunodetection Kit, Invitrogen). Nonspecific reactivity was blocked by incubation with the blocking reagent supplied in the kit. Membranes were further treated by incubating with primary antibodies (Table 1) for 2 h at room temperature or overnight at 4°C, followed by incubation for 30 min at room temperature with appropriate secondary anti-rabbit antibody supplied in the kit. Bands were visualized with the 5-Bromo-4-chloro-3-indolyl phosphate/nitroblue tetrazolium chloride based chromogenic substrate. For loading control, immunoblotting of the same lysates was performed using either Beta-actin (*β*-actin) antibody (Abcam Bioscience, UK) or the PVDF membranes with transferred proteins visualised using Ponceau stain (Sigma).

### 2.5. Luciferase Reporter Assay

For the analysis of promoter activities and transcriptional regulation, the 1.5 kb promoter regions of* HER2* and* HER3* genes cloned in pGL3 basic vector (Promega) were transfected into relevant cell lines. Briefly, cells were seeded in triplicate in 24-well plates at a density of 2 × 10^5^ cells per well and allowed to attach for 18 h. Following this, cells were transfected with either 1 *μ*g of empty pGL3 basic vector (Promega) or pGL3 basic vector with cloned fragments of HER2 or HER3 promoters driving the expression of luciferase gene, using Lipofectamine 3000 as transfection reagent according to manufacturer's instructions (Life Technologies). Cotransfection was also performed with 0.2 *μ*g of pRL-CMV vector (Promega) to provide for an internal control of transfection. Following this, cells were allowed to grow for 24 h, subjected to desired treatments and lysed and protein lysates transferred to opaque white bottom 96-well plates. The dual luciferase activity of fire fly luciferase (from cloned promoters) and Renilla (internal control) in the harvested lysates was measured sequentially by following manufacturer's instructions (Promega) and taking luminescence readings in luminometer (MODULUS, Promega). To determine the transcriptional activity of NRF2 in PEO1 and SKOV3 cell lines, basic pGL3 vector (Promega) containing cloned 8 x* cis* regulatory ARE promoter elements was transfected into the cell lines grown in 24-well plates and subjected to dual luciferase reporter assay (Promega) as described above.

### 2.6. SiRNA Transfection

Small inhibitory RNA (SiRNA) was used to knockdown NRF2 (Hs_NFE2L2_6, Qiagen). For SiRNA transfection, cells were seeded in triplicate either in 24-well plates (0.5 × 10^5^ cells), in 60 mm plates with cells grown on poly-L lysine coated coverslips (0.5 × 10^6^ cells), or in 96-well plates in triplicate (2 × 10^4^) and allowed to grow for 24 h. Following this, cells were cotransfected using either 20 pmol SiRNA and 1 *μ*g of different PGL3 promoter constructs (24-well plate) or 75 pmol and 100 pmol SiRNA only (60 mm plates) or 7 pmol of SiRNA (96-well plate) and incubated for a further 24 h. Cells transfected in 24-well plate were further processed for dual luciferase assay and those in 60 mm plates were harvested for immunoblotting or used for imaging analysis while those in 96-well plates were processed for cytotoxicity assay. In all cases, scrambled SiRNA was used as a control while transfection was performed using Lipofectamine 3000 (Life Technologies) according to manufacturer's instructions.

### 2.7. Cytotoxicity Assay

For cytotoxicity (or cell viability) assay, cells were seeded in triplicate at a density of 0.5 × 10^4^ cells in 96-well plate and allowed to attach for 18 h. On the day of treatment, old media were removed and 80 *μ*L of media containing relevant drugs was added and the plate was incubated for the required period of time. On the day of assay, 20 *μ*L of the 5 mg/mL MTT stock was added to each well and plate was further incubated for 4 h. Following this, the old media with MTT were removed, cells were gently washed with prewarmed PBS, and 100 *μ*L of DMSO was added to solubilise the internalised MTT by shaking over an orbital shaker for 15 min. Absorbance of the released dye was measured and recorded using multiplate reader (MODULUS, Promega) at 540 nm.

### 2.8. Immunocytochemistry/Immunolabelling

For immunocytochemistry, exponentially growing cells were seeded at a density of 5 × 10^4^ cells in complete media onto poly-L lysine (Sigma-Aldrich) coated cover slips placed in a 12-well tissue culture plates. Next day, following relevant treatments, cells were washed three times with ice cold PBS and fixed in 3.5% paraformaldehyde in a standard PBS at room temperature for 30 min. Following this, cells were gently washed twice with 1 mL of PBS, permeabilized with 0.3% Triton X-100 for 10 min, and, following three washes with PBS, blocked with a solution containing 1% goat serum, 1% bovine serum albumin, and 0.05% Triton X-100 in PBS for 30 min. Cells were then incubated with relevant primary antibody (Table 1) diluted in blocking solution for 1 h, washed three times with 0.1% Triton X-100/PBS for 5 min, and then incubated with Alexa Fluor 488 or 568 conjugated goat anti-rabbit (Table 1) for 30 min. After subsequent three washes with the 0.1% Triton X-100 in PBS for 5 min, cover slips with cells were mounted on slide using 4′,6-Diamidino-2-Phenylindole, Dihydrochloride (DAPI) containing mounting reagent (Life Technologies) and imaged under relevant filters with a Leica DMiRe2 electronic microscope.

### 2.9. Statistical Analysis

All statistical analyses were performed using statistical software SPSS (IBM, version 22). Test for normality of data was determined by Shapiro-Wilk and Kolmogorov and Smirnov tests. The significance (*p* value) of differences of pooled results was determined by either independent *t*-tests or One WAY ANOVA followed by post hoc Tukey's tests. Significance was defined as *∗* = *p* < 0.05, *∗∗* = *p* < 0.01, and *∗∗∗* = *p* < 0.001.

### 2.10. Imaging and Analysis

Quantitative analysis of raw immunoblots was performed by capturing the images in high resolution TIFF format files using a charge-coupled-device camera (AxioCam MRc, Carl Zeiss) and subjected to Gelpro analysis software, version 3.1 (Gelpro Media Cybernetics) for integrated optimal densitometry. Fluorescence images of immunocytochemistry were collected under relevant excitation and emission filters depending on the fluorotype under Leica DMiRe2 electronic microscope equipped with iXonEM +897 EMCCD camera (ANDOR Technologies Ltd.). Images were analysed using multidimensional microscopy software Andor Module iQ Core. Colocalization assay was performed and determined with software integral features supplied by Andor iQ Core software. Data were generally expressed as mean ± S.D. for individual sets of experiments.

## 3. Results

### 3.1. Pharmacological Activation of NRF2 Enhances Ovarian Cancer Cell Growth and Protects from Cytotoxicity Caused by HER2-Targeted Immunotherapeutic Agents

**Figure 1:**
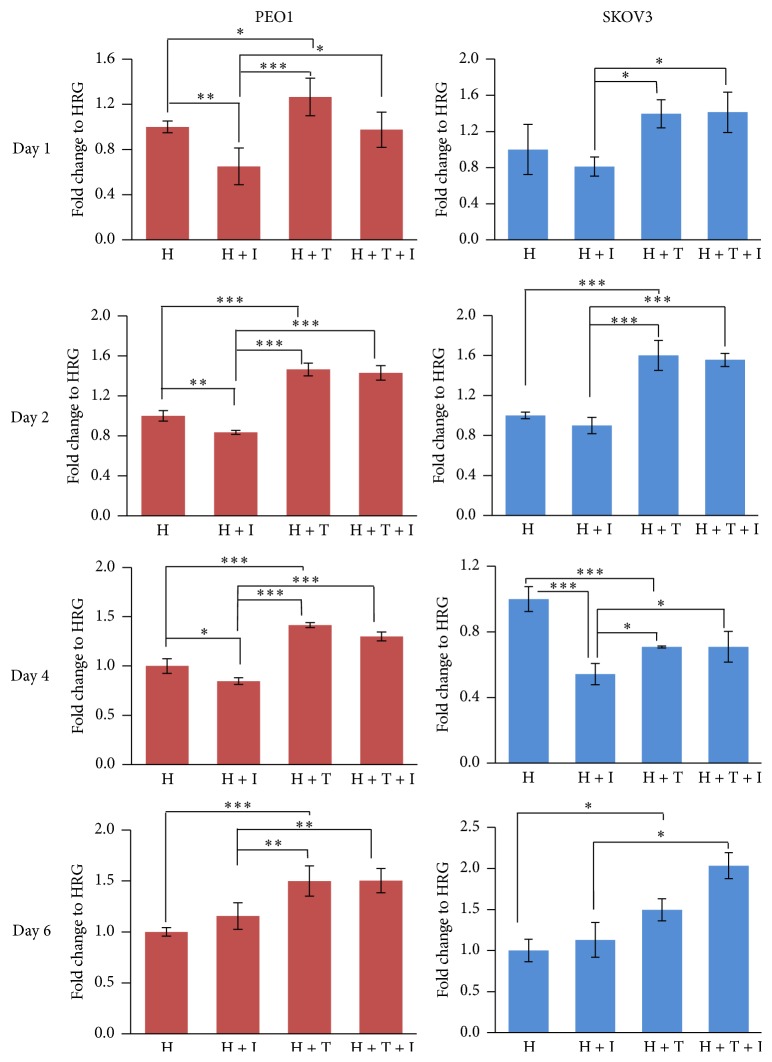
NRF2 activation causes cytoprotection from HER2-targeted agents, Pertuzumab and Trastuzumab. PE01 or SKOV3 cells in the presence of 1 nM HRG were either left untreated (H) or treated with 20 *μ*g/mL of HER2 inhibitors Pertuzumab and Trastuzumab (H + I), 200 *μ*M tBHQ (H + T), or combination of inhibitors and tBHQ (H + T + I). TBHQ was added 5 h in advance. Cell number was assessed indirectly by use of the MTT assay. Values shown are means ± S.D. of triplicates normalised to untreated controls expressed as 1. Statistical significance was calculated between H + I, H + T, and H + T + I groups by ONE WAY ANOVA followed by Tukey's post hoc test according to the scale *∗*:  *p* < 0.05, *∗∗*:  *p* < 0.01, and *∗∗∗*:  *p* < 0.001.

Numerous studies have shown that NRF2 promotes resistance to chemotherapeutic agents [50, 51] and contributes to general cytoprotection, metabolic reprograming, and cell survival [52–55]. On the other hand, targeted immunotherapy involving inhibitory monoclonal antibodies against HER2 receptor has generated interest in recent years as a potential strategy to overcome ovarian cancer cell therapeutic resistance [17, 56]. Using HER2 overexpressing and low expressing ovarian cancer cell lines SKOV3 and PEO1, respectively [57], we first examined whether preactivation of NRF2 would change the cytotoxic responses of these cells to HER2-targeted immunotherapeutic agents Pertuzumab and Trastuzumab. For this, cells were grown in media containing 5% charcoal stripped FBS and 1 nmol/L Heregulin (HRG), a ligand for the HER3 receptor [58] for relevant treatments. Firstly, we found that pharmacological activation of NRF2 by tBHQ alone was sufficient to enhance the proliferation of both cell lines for six days (Figure 1). On the other hand and as expected, exposure of cells to HER2 inhibitors, Pertuzumab and Trastuzumab, inhibited the proliferation of both cell lines for up to 4 days of treatment, while losing its inhibitory effect on day 6. Interestingly, pretreatment of cells with 200 *μ*M tBHQ for 5 h before the introduction of the HER2 inhibitors significantly protected cells from the inhibitory action of the subsequently added HER2 targeting monoclonal antibodies. This was consistent for both cell lines and for all the treatment days tested (Figure 1). Furthermore, inclusion of tBHQ with the inhibitors not only protected the cells but increased survival even beyond the untreated levels on days 2, 4, and 6 in PEO1 and days 1, 2, and 6 in SKOV3 cell lines (Figure 1). This demonstrated that NRF2 activation is not only implicated in resistance to genotoxic agents as previously demonstrated [55] but can also lead to resistance to immunotherapies involving Pertuzumab and Trastuzumab whose actions otherwise are very specific to HER2 receptors and unrelated to antioxidant pathway.

### 3.2. TBHQ Treatment Causes Protein Induction of HER2 and HER3 and Parallel Increase in Phospho-AKT S473

**Figure 2:**
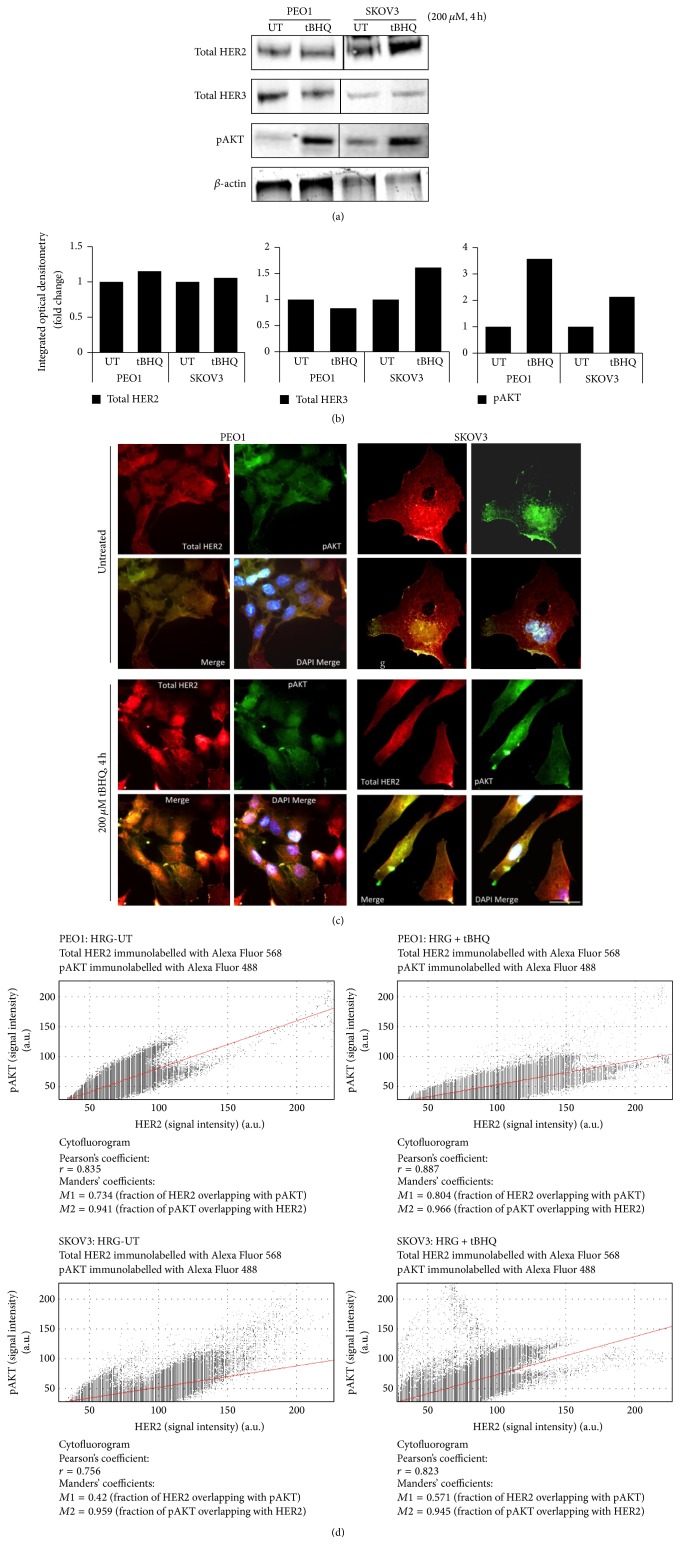
TBHQ treatment causes protein induction of HER2 and HER3 and upregulation of pAKT levels in PEO1 and SKOV3 cells. (a) Immunoblot analysis following treatment with tBHQ demonstrated protein induction of both HER2 and HER3 receptors and increase of pAKT. Exponentially growing cells were either left untreated (UT) or treated with 200 *μ*M tBHQ for 4 h before being harvested and processed for immunoblotting using relevant antibodies (Table 1). (b) Bar chart showing total HER2, total HER3, and phospho-AKT levels in PEO1 and SKOV3 cell lines by quantifying immunoblot signal intensities obtained in (a) and normalised to the value of UT and expressed as fold change. (c) Immunofluorescent labelling of endogenous HER2 and phospho-AKT reveals protein induction following tBHQ treatment. Cells were processed for immunocytochemistry and immunolabelled using anti HER2 (red fluorescence) or phospho-AKT (green fluorescence) primary antibodies followed by Alexa Fluor conjugated secondary antibodies. Nuclear reference was provided by costaining with 4′,6-Diamidino-2-Phenylindole, Dihydrochloride (DAPI). Scale bar indicates 10 *μ*m. (d) Analysis of colocalisation between immunostained HER2 and pAKT in the images obtained in (c). Spatial correlation between the two fluorescent signals was obtained by generating cytofluorograms and performing Pearson's correlation analysis.

Previous studies have examined the crosstalk between growth promoting MAPK and PI3K pathways and NRF2 antioxidant pathway in numerous cell systems. However, in the majority of such studies, the focus was regulation of NRF2 by these kinases [46, 47, 59, 60]. The observation that preactivation of NRF2 led to resistance against agents of targeted therapy (Figure 1) suggested potential regulation of HER2 dependent growth pathways by NRF2. Hence, we next exposed PEO1 and SKOV3 cell lines to a single concentration of tBHQ for 4 h and examined the effects of such treatment on the protein levels of HER2 and HER3 and their downstream substrate pAKT Ser 473. Firstly, higher levels of HER2 receptor were confirmed in SKOV3 cell line that was also accompanied by induced basal pAKT, consistent with previous reports [61]. Secondly, following tBHQ treatment, we saw induction of total HER2 and parallel consequential induction of pATK levels in both cell lines. Further, HER3 was found to decrease in PEO1 while being induced in SKOV3, demonstrating a differential regulation of the receptors in the two cell lines (Figures 2(a) and 2(b)). This is consistent with the increased proliferation seen following tBHQ treatment, as an enhanced cell surface expression of receptors would lead to a greater degree of binding to their HRG ligand and triggers growth promoting signaling.

To explore further HER2 and pAKT induction by tBHQ at a single cell level, we performed subcellular localisation by fluorescent double immunolabelling of these proteins in PEO1 and SKOV3 cell lines following the same treatments (Figure 2(c)). Consistent with Figure 2(a), we saw higher expression of HER2 in SKOV3 as compared to PEO1. For both cell lines, pAKT was found uniformly distributed in the cytoplasm and nucleus. This could be indicating the constitutively active nature of this pathway and could be explained by the presence of HRG in the media. Following tBHQ treatment for 4 h, we saw an increase in HER2 expression and an accompanying increase in pAKT levels as well. Superimposition and colocalisation of the images captured in the red and green fluorescence channels to indicate HER2 and pAKT, respectively, were performed and showed increased localisation of the two proteins, as demonstrated by the appearance of yellow fluorescence following tBHQ as compared to untreated controls (Figure 2(c)). To confirm and measure the enhanced colocalisation following treatments, we also performed further imaging analysis by generating cytofluorograms and found that Pearson's coefficient of correlation (*r*) increased in both cell lines following tBHQ exposure (Figure 2(d)). Altogether, these data illustrated effects of tBHQ treatment on RTK mediated growth signaling. This was demonstrated by induction of HER2 and HER3 and activation of AKT following pharmacological activation of NRF2, which supported the enhanced proliferation seen before (Figure 1).

### 3.3. Pharmacological Activation of NRF2 Causes Transcriptional Induction of* HER2* and* HER3* Genes

**Figure 3:**
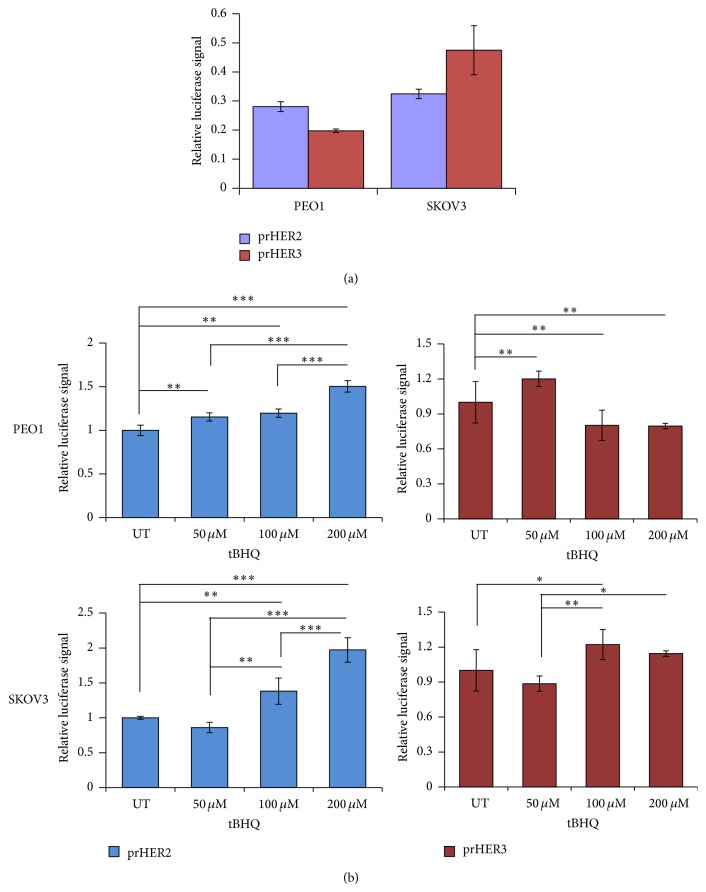
Nrf2 activation leads to transcriptional induction of HER2 and HER3 in a concentration dependent manner. (a) SKOV3 cells exhibit higher basal transcription of both* HER2* and* HER3*. PEO1 and SKOV3 cells were transfected with either empty PGL3 basic vector or 1 *μ*g PGL3 basic vector with cloned 1.5 kb fragments of either HER2 (prHER2) or HER3 (prHER3) promoter driving the expression of luciferase gene. Cotransfection with 0.2 *μ*g pRL-CMV plasmid was performed as an internal transfection control. (b) TBHQ causes transcriptional induction of HER2 and HER3 in a concentration dependent manner. PEO1 and SKOV3 cell lines were transfected in triplicate as in (a) but were treated with different concentrations of tBHQ as indicated for 4 h. Data shown are the means ± S.D. of triplicates, normalised to untreated (UT) controls and expressed as fold change with statistical significance determined by ONE WAY ANOVA followed by Tukey's post hoc test (*∗*:  *p* < 0.05, *∗∗*:  *p* < 0.01, and *∗∗∗*:  *p* < 0.001).

Previous studies have shown transcriptional perturbation of HER2 and HER3 following different targeted therapy treatments [17]. In some contexts, this was proposed to be used as a biomarker for treatment response [61]. After finding the modulatory effects of tBHQ treatment on protein levels of HER2 and HER3 receptors, we next wanted to identify the mechanism of this upregulation. Specifically, we wanted to examine whether the protein inductions seen in Figure 2 result from transcriptional regulation. To determine this, we generated transcriptional reporter assays for both HER2 and HER3 receptors. This involved developing HER2 and HER3 promoter driven luciferase reporter system (named prHER2 and prHER3, resp.). We transfected these luciferase reporter systems carrying 1.5 kb of the upstream promoter regions of the two receptors into both PEO1 and SKOV3 cell lines to first determine their basal level of transcription and then studied the effects of tBHQ treatment.  Figure 3(a) interestingly revealed that SKOV3 cell line exhibited enhanced basal transcription of both* HER2* and* HER3* genes as compared to PEO1 cell line (Figure 3(a)). However, the previous western blot analysis in Figure 2 showed higher basal levels of HER3 in PEO1 as compared to SKOV3 whereas HER2 levels were consistently higher in SKOV3. This illustrated that the overexpression of HER2 in SKOV3 cell line could be explained by both gene amplification [62] and higher basal transcription.

We next exposed cells transfected with the prHER2 and prHER3 reporter assays to increasing concentrations of tBHQ to further explore the nature of this transcriptional regulation. Strikingly, both PEO1 and SKOV3 cell lines exhibited significant dose-dependent transcriptional induction of HER2 (Figure 3(b), blue bars). Interestingly, prHER3 exhibited a varying response. While prHER3 activity was significantly induced in PEO1 following 50 *μ*m tBHQ, increasing dosage beyond 50 *μ*M led to its repression. In SKOV3 cells on the contrary, 50 *μ*M tBHQ repressed prHER3 activity while increasing dosage of 100 and 200 *μ*M led to subsequent induction (Figure 3(b), brown bars). This complex regulation of HER3 is reminiscent of recent reports that revealed that induction of HER2 might repress HER3 expression while its inhibition could lead to transcriptional induction of HER3 [13, 14, 63]. This set of results confirmed the transcriptional basis of induction of HER2 and HER3 protein levels, which concomitantly also led to pAKT induction.

### 3.4. NRF2 Activation Desensitises RTK Signaling Pathway to Combination of HER2 Targeting Monoclonal Antibodies Pertuzumab and Trastuzumab

**Figure 4:**
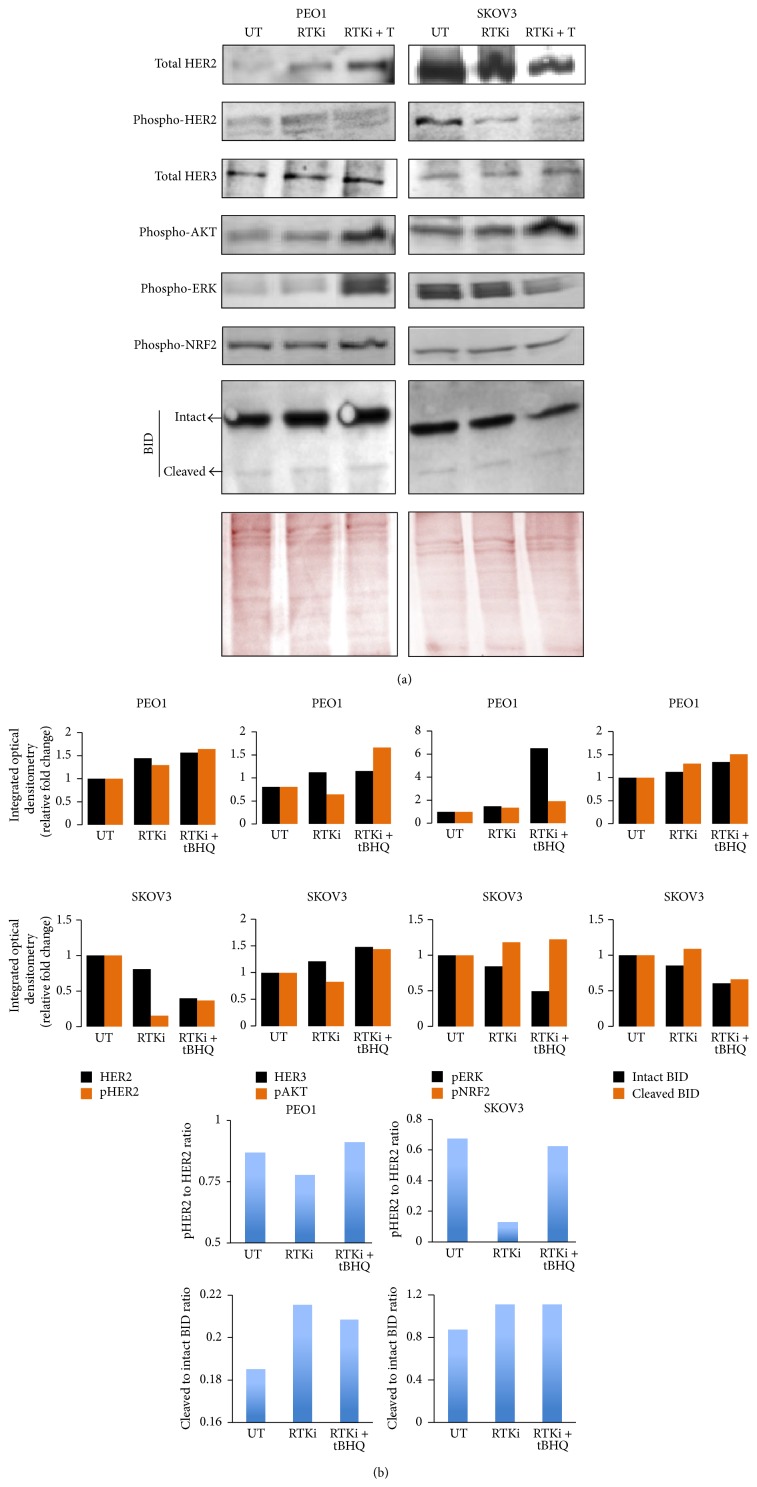
NRF2 activation desensitises RTK signaling pathway to HER2 inhibitors Pertuzumab and Trastuzumab. (a) Immunoblot analysis showing tBHQ dependent recovery of RTK signaling following its inhibition by HER2 inhibitors. Exponentially growing cells were either left untreated (UT) or treated with combination of HER2 inhibitors, Pertuzumab and Trastuzumab at concentration of 20 *μ*g/mL (RTKi), or with cotreatment of 200 *μ*M tBHQ (RTKi + T) for 4 h before and processed for immunoblotting using relevant antibodies (Table 1). Ponceau stain of the same blot was used as loading control. (b) Bar chart showing total HER2, phospho-HER2, total HER3, phospho-AKT, phospho-ERK, phospho-NRF2, and BID levels in PEO1 and SKOV3 cell lines by quantifying immunoblot signal intensities obtained in (a) and normalised to the value of UT and expressed as fold change. Blue bars show ratio of phospho-HER2 to HER2 (upper panels) and cleaved BID to intact BID (lower panels).

The observation that tBHQ treatment led to transcriptional induction of HER2 and HER3 suggests that NRF2 may be directly involved in regulating the receptor expression and as such may influence responses to targeted therapies involving HER2 inhibitors. This important question was next investigated by treating PEO1 or SKOV3 cells either with the combination of Pertuzumab and Trastuzumab alone or by cotreatment with tBHQ to examine the consequences of NRF2 activation on drug responses. Interestingly, some features of the signaling response were similar between these two cell lines while others were more distinct. In the PEO1 cell line, treatment with inhibitors alone induced both HER2 and HER3 levels consistent with the parallel increase in phospho-HER2 T877 (Figure 4) in this cell line. In contrast, for SKOV3, only HER3 expression showed a minor induction while total HER2 levels were reduced explaining the decrease in phospho-HER2 T877 levels as well. In order to better understand the effect of these inhibitors on RTK signaling, we normalised the blot signal of phospho-HER2 in both cell lines to the corresponding values of total HER2 (Figure 4, blue bars). This analysis interestingly revealed that while the inhibitors reduced the ratio of phospho-HER2 to total HER2, cotreatment with tBHQ restored the ratio back to that of untreated controls. This effect was more pronounced in the SKOV3 cell line. Importantly, in terms of pAKT S473, while 4 h treatment with inhibitors led to minor repression of its levels as revealed by the densitometry analysis, cotreatment with tBHQ protected this repression and increased pAKT levels beyond that of untreated controls (Figures 4(a) and 4(b)). These results revealed that tBHQ can protect RTK signaling against the inhibitory action of the drugs. We also included phospho-ERK p44/p22 levels in our analysis as ERK was previously shown to be inhibited by drugs targeting HER2 receptor [56]. We saw a very minor repression of phospho-ERK levels only in SKOV3 cells following 4 h of inhibitor treatment. However, tBHQ dependent induction for ERK was seen in PEO1 cells. Inhibitor treatment did not influence pNRF2 S15 levels in either of the cell lines, but as expected, its levels increased following tBHQ treatment. Finally, we examined intact and cleaved levels of proapoptotic protein BID in order to further support our conclusions drawn from Figure 1. By determining the ratio of cleaved BID over intact, we observed that while treatment with inhibitors induced levels of cleaved BID, tBHQ cotreatment led to a minor repression, further explaining the cytoprotective effect of tBHQ treatment (Figure 4). These results revealed important consequences of tBHQ treatment on targeted therapy using HER2-targeted monoclonal antibodies and showed that treatment with NRF2 activator attenuated the inhibitory action of these monoclonal antibodies.

### 3.5. Knockdown of NRF2 by Small Inhibitory RNA (SiRNA) Elevates ROS, Represses pNRF2 and Heme Oxygenase-1 (HO-1) Levels, and Disrupts tBHQ Dependent Induction of ARE

**Figure 5:**
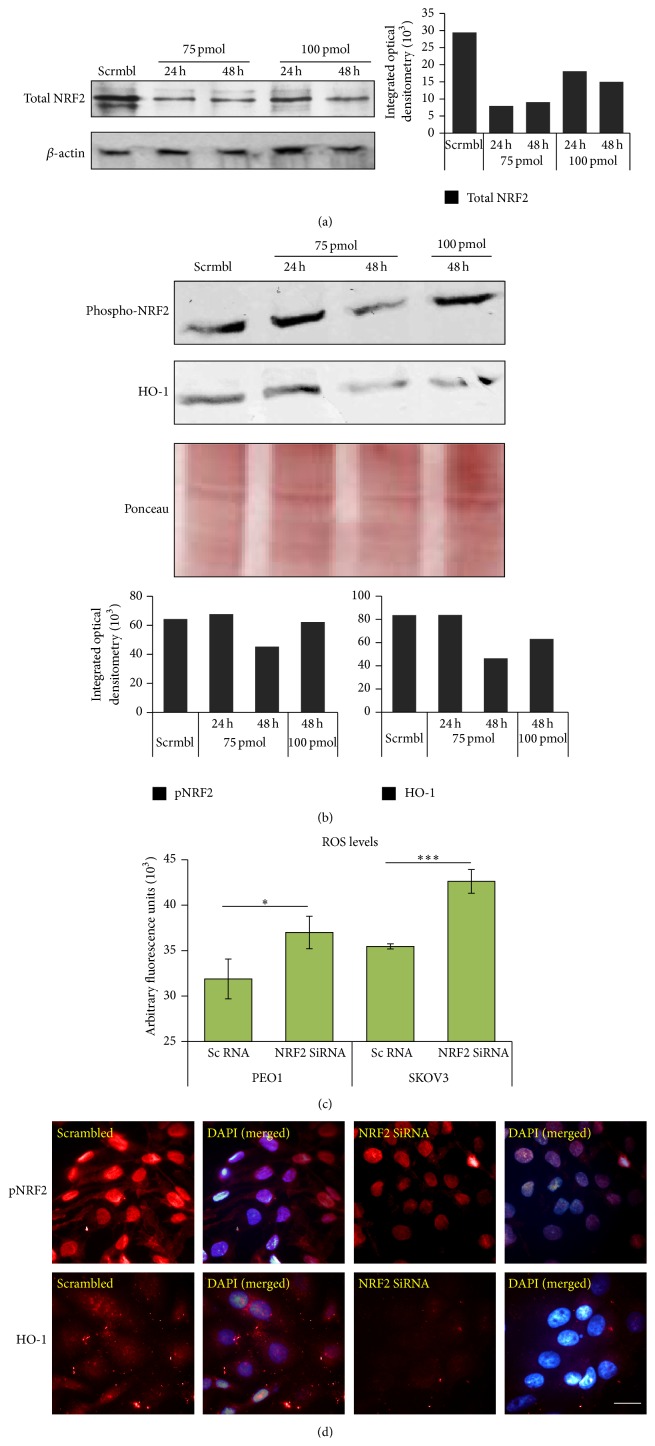
Knockdown of NRF2 by SiRNA causes repression of phospho-NRF2 and HO-1 levels and elevation of reactive oxygen species (ROS). (a) Optimization of SiRNA-mediated NRF2 knockdown. Exponentially growing cells were transfected either with scrambled RNA (scrmbl) or with different amounts of SiRNA for either 24 h or 48 h before being processed for immunoblotting. (b) NRF2 knockdown results in repression of its substrates. The same lysates as in (a) were blotted for phospho-NRF2 and HO-1 levels. Bar charts in (a) and (b) show total NRF2, phospho-NRF2, and HO-1 levels in SKOV3 cell lines by quantifying immunoblot signal intensities obtained in respective blots and normalised to the value of UT and expressed as fold change. (c) NRF2 knockdown leads to ROS accumulation. SKOV3 cells were seeded in triplicate for 18 h and transfected with NRF2 SiRNA. Following 48 h incubation, cells were assayed for total ROS by loading them with DCFDA for 45 min and measuring fluorescence using fluorescence multiplate reader (MODULUS, Promega) with excitation and emission spectra of 485 nm/535 nm. The fluorescence reading was normalised to total cell abundance within the same wells as described in Materials and Methods. Data are the means with ±S.D. of triplicates, normalised to untreated (UT) control and expressed as fold change with statistical significance determined by Student's *t*-test according to the scale *∗*:  *p* < 0.05, *∗∗*:  *p* < 0.01, and *∗∗∗*:  *p* < 0.001. (d) Immunofluorescent labelling of endogenous phospho-NRF2 and HO-1 exhibits repression following NRF2 knockdown. Cells were transfected as in (a) and processed for immunocytochemistry. Relevant primary antibodies followed by Alexa Fluor conjugated secondary antibodies were used for immunolabelling for phospho-NRF2 and HO-1 (red fluorescence). Nuclear reference was provided by costaining with 4′,6-Diamidino-2-Phenylindole, Dihydrochloride (DAPI). Images were captured with Leica DMiRe2 electronic microscope with 100x objective while merging, colocalisation, and further analysis were performed by using integrated features of Andor iQ Core software (ANDOR Technologies Ltd.). Scale bar indicates 10 *μ*m.

**Figure 6:**
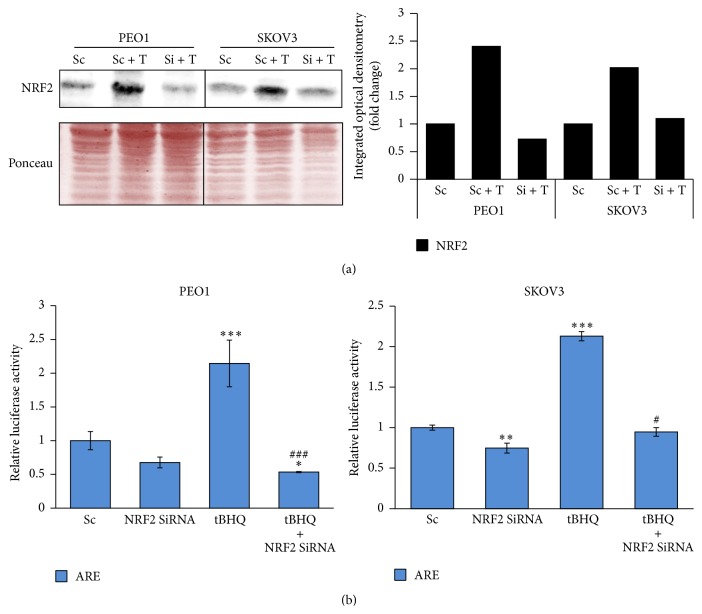
Knockdown of NRF2 by SiRNA represses both basal and induced antioxidant response pathway in PEO1 and SKOV3 cell lines. (a) Immunoblotting analysis showing repression of NRF2 following NRF2 knockdown by SiRNA in PEO1 and SKOV3 cell lines. Cells were either transfected with scrambled SiRNA (Sc) or transfected with 75 pmol of NRF2 SiRNA (Si). After 48 h, cells were either left untreated or treated with 200 *μ*M tBHQ (T) for 4 h, before being processed for immunoblotting using relevant antibodies (Table 1). Ponceau stain of the same blot was used as loading control. Bar chart shows NRF2 levels by quantifying immunoblot signal intensities obtained in (a) and normalised to the value of untreated (UT) control and expressed as fold change. (b) Knockdown of NRF2 causes inhibition of its transcriptional antioxidant program in both constitutive and tBHQ induced states. PEO1 and SKOV3 cells were transfected with either empty PGL3 basic vector or 1 *μ*g PGL3 basic vector with a cloned 8 x* cis*-antioxidant response elements (ARE) driving NRF2 dependent expression of luciferase gene. Cotransfection with 0.2 *μ*g pRL-CMV plasmid was performed as an internal transfection control. Where required, cotransfection with either scrambled RNA (Sc) or NRF2 SiRNA was performed using 20 pmol SiRNA. At 24 h after transfection, treatment with 200 *μ*M tBHQ was performed where indicated for 4 h following which, cells were processed for dual luciferase reporter assay (Promega) to record luciferase activity in multiplate reader (MODULUS, Promega). Data are the means with ±S.D. of triplicates normalised to the value of scrambled SiRNA (Sc) and expressed as fold change with statistical significance determined by ONE WAY ANOVA followed by Tukey's post hoc test. *∗* indicates significance of scramble versus treatment groups while # indicates significance of tBHQ versus tBHQ + NRF2 SiRNA groups according to the scale symbolised by *∗* or #:  *p* < 0.05, *∗∗* or ##:  *p* < 0.01, and *∗∗∗* or ###:  *p* < 0.001.

In order to confirm the direct role of NRF2 in tBHQ dependent induction of HER2 and HER3 receptors, we next knocked down NRF2 using SiRNA. To this end, we first optimized and verified sufficient knockdown of NRF2 using specific SiRNA and then studied the effects of this knockdown on antioxidant pathway. As shown in Figure 5(a), 75 pmol of NRF2 SiRNA produced maximum depletion of NRF2 both following 24 and 48 h of transfection, while 100 pmol showed lesser depletion (Figure 5(a), black bars indicating band intensities). We next determined whether this depletion is sufficient to cause repression of the antioxidant pathway by examining NRF2 substrates. We found that 75 pmol SiRNA sufficiently downregulated phospho-NRF2 and HO-1 levels as well (Figure 5(b)). Efficiency of internalisation of NRF2 targeting SiRNA in SKOV3 cells using different amounts was confirmed and verified (Figure 5(c)). We next quantified total basal ROS following NRF2 knockdown to determine whether NRF2 depletion caused elevation of ROS. Loading of cells with 2′,7′-Dichlorofluorescin diacetate dye which is a fluorescent marker of intracellular ROS confirmed elevation of ROS resulting from NRF2 knockdown (Figure 5(c)). Finally, we performed immunostaining of endogenous pNRF2 and HO-1 following transfection with either scrambled or NRF2 targeting SiRNA and as consistent with Figure 5(b), we verified repression of pNRF2 and HO-1 levels at single cell level (Figure 5(d)). Having confirmed the effectiveness of our SiRNA-mediated NRF2 knockdown, we next examined whether depletion of NRF2 would also disrupt tBHQ dependent induction of antioxidant pathway in PEO1 and SKOV3 cell lines and thus confirm the direct involvement of NRF2 in this mechanism. To do this, we exposed cells to tBHQ either in the presence of endogenous NRF2 or following its genetic depletion.  Figure 6(a) revealed that NRF2 protein induction seen in tBHQ treatment was disrupted following its SiRNA transfection. Next, in order to confirm that NRF2 depletion also caused inhibition of transcriptional antioxidant response program and to further confirm the conclusions drawn from Figure 5(b), we transfected cells with* cis-*antioxidant response elements (ARE) in luciferase reporter vector driving the expression of luciferase to report transcriptional activity of NRF2. Firstly, we saw repression of ARE signal supporting our conclusions drawn from Figure 5. Secondly, we saw that the tBHQ treatment regime that had caused induction of NRF2 protein levels (Figure 6(a)), and those of NRF2 substrate HO-1 (Figure 5(b)) and HER2 and HER3 receptor expressions (Figures 2 and 3), also significantly enhanced the activity of the NRF2 dependent antioxidant transcriptional programme in both PEO1 and SKOV3 cell lines (Figure 6(b)). Finally, we saw that such induction was inhibited following knockdown of NRF2 to significant levels as compared to tBHQ treatment alone. Altogether, Figures 5 and 6 provide evidence of knockdown of NRF2, repression of the antioxidant response pathway, and disruption of tBHQ mediated pathway induction.

### 3.6. NRF2 Depletion Causes Transcriptional Inhibition of* HER2* and* HER3* Leading to Repression of HER2, HER3, and pAKT Proteins and Sensitisation to Targeted Immunotherapeutics

**Figure 7:**
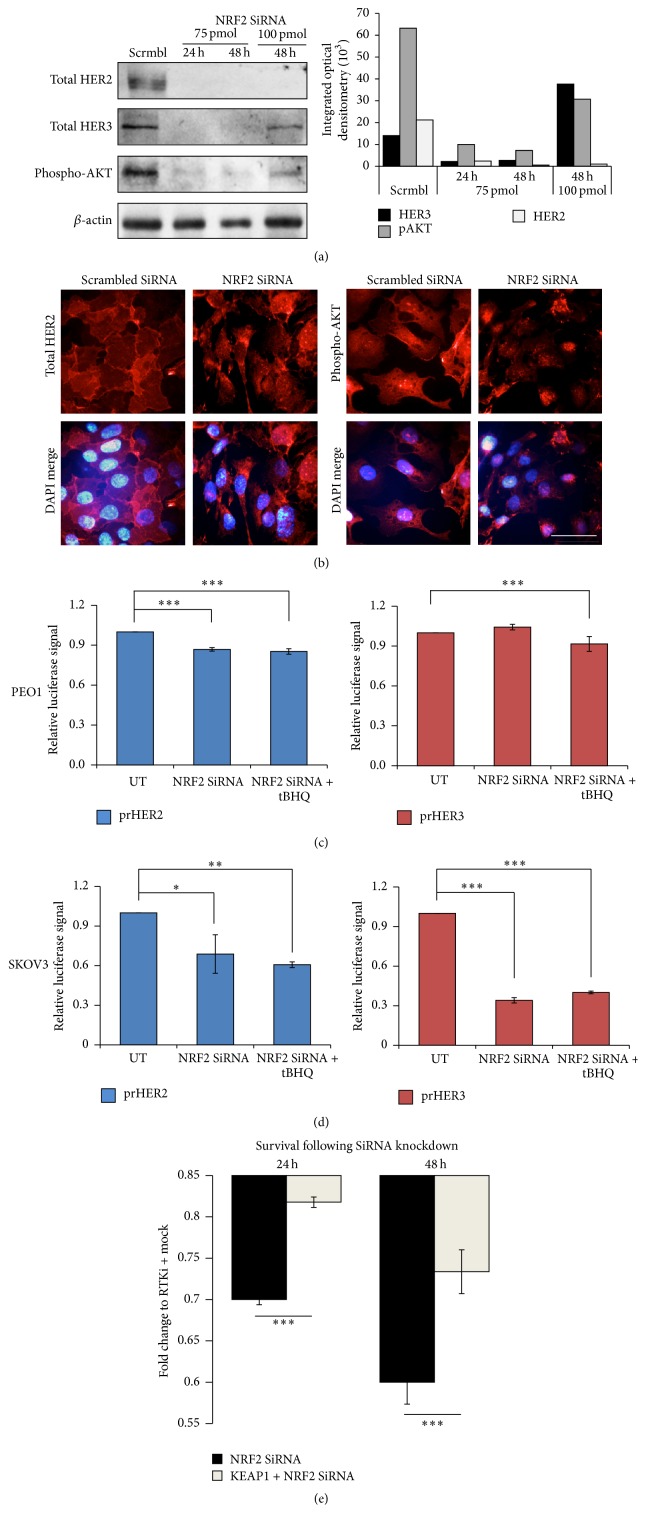
NRF2 knockdown causes downregulation of HER2 and HER3 levels, repression of pAKT, and sensitisation to targeted immunotherapeutics. (a) Immunoblotting analysis showing inhibition of RTK signaling following depletion of NRF2 mRNA by SiRNA in SKOV3 cell line. Exponentially growing cells were either transfected with scrambled SiRNA (Scrmbl) or transfected with 75 pmol of NRF2 SiRNA for either 24 or 48 h or 100 pmol of NRF2 SiRNA for 48 h and processed for immunoblotting using relevant antibodies (Table 1). *β*-actin was used as a loading control. Bar chart shows protein levels by quantifying immunoblot signal intensities obtained and normalised to the value of untreated (UT) control and expressed as fold change. (b) Immunofluorescent labelling of endogenous total HER2 or phospho-AKT exhibits repression following NRF2 knockdown. Cells were transfected as in (a) and processed for immunocytochemistry. Relevant primary antibodies were used to stain HER2 or phospho-AKT followed by Alexa Fluor conjugated secondary antibody (red fluorescence). Nuclear reference was provided by costaining with 4′,6-Diamidino-2-Phenylindole, Dihydrochloride (DAPI). Images were captured with Leica DMiRe2 electronic microscope at 100x objective while merging, colocalisation, and further analysis were performed by using integrated features of Andor iQ Core software (ANDOR Technologies Ltd.). Scale bar indicates 10 *μ*m. (c and d) HER2 and HER3 downregulation following NRF2 knockdown is caused by their transcriptional repression. Exponentially growing PEO1 cells (c) or SKOV3 cells (d) were transfected with either empty PGL3 basic vector or 1 *μ*g PGL3 basic vector with cloned 1.5 kb fragments of either HER2 (prHER2) or HER3 (prHER3) upstream promoter regions driving the expression of luciferase gene. Cotransfection with 0.2 *μ*g pRL-CMV plasmid was performed as an internal transfection control. At 24 h after transfection, cells were either left untreated (UT) or treated with 200 *μ*M tBHQ as indicated for 4 h following which, cells were processed for dual luciferase reporter assay (Promega) to record luciferase activity in multiplate reader (MODULUS, Promega). (d) The same was done for SKOV3 cell lines. (e) Knockdown of NRF2 through SiRNA sensitises cancer cell to RTK inhibitors while parallel knockdown of KEAP1 partially relieves this sensitisation. Cells were transfected with scrambled SiRNA or SiRNA targeting NRF2 either alone or with the inclusion of KEAP1 SiRNA. Following further 24 h incubation, cells were either left untreated or treated with 25 *μ*g/mL of HER2 inhibitors Pertuzumab and Trastuzumab. Cytotoxicity assay was performed as in (a). In (c–e), data are the means with ±S.D. of triplicates and expressed as fold change with statistical significance determined by ONE WAY ANOVA followed by Tukey's post hoc test (for c and d), or Student's *t*-test (for e) according to the scale *∗*:  *p* < 0.05, *∗∗*:  *p* < 0.01, and *∗∗∗*:  *p* < 0.001.

As shown in the previous sections, NRF2 activation by tBHQ not only induced the NRF2 dependent antioxidant response pathway as expected, but surprisingly also induced protein levels of HER2 and HER3 (Figure 2) and we further confirmed that the protein upregulation was as a result of their transcriptional induction (Figure 3). These findings were important because such receptor induction attenuated the inhibitory responses of HER2-targeted drugs (Figure 4). As an alternative approach to study the regulation of HER receptors by NRF2, we knocked down NRF2 in our cell lines and firstly studied the protein levels of the receptors and their downstream substrate, pATK. We found significant protein repression of HER2 and HER3 as well as pAKT. Quantification of the resulting immunoblot signals revealed a greater repression with 75 pmol NRF2 SiRNA (Figure 7(a)). Interestingly, we could also detect and capture such repression at single cell level by performing immunostaining for HER2 and pAKT following either scrambled or NRF2 specific SiRNA (Figure 7(b)). Immunolabelling also revealed localisational features of total HER2 and pAKT. HER2 was mostly localised at the cell membrane as expected and apparently without any nuclear staining. The pAKT on the other hand was localised at the cell membrane, general cytosol and nucleus, as revealed by immunostaining and the merger with DAPI staining (Figure 7(b)). This is consistent with previous reports of nucleocytoplasmic shuttling of pATK that could have physiological consequences [63–65].

We next wanted to determine and confirm any transcriptional mechanism of NRF2 specific SiRNA dependent repression of HER2 and HER3. We thought this could be a likely explanation as we earlier showed tBHQ dependent transcriptional upregulation of HER receptors (Figure 3). To address this, we again utilised our transcriptional reporter assays for both HER2 and HER3 receptors that were established for this study. Using our ovarian cell line models, we individually transfected the reporter systems but, this time, cotransfecting with NRF2 specific SiRNA as well. Following this, cells were either left untreated or treated with tBHQ. We found that following NRF2 knockdown, HER2 transcription was significantly repressed in both PEO1 (Figure 7(c)) and SKOV3 (Figure 7(d)) cell lines. In terms of HER3, while NRF2 knockdown significantly repressed transcription in SKOV3, such repression was not seen in PEO1 (compare Figures 7(c) and 7(d) for prHER3). Interestingly, the tBHQ dependent transcriptional induction of prHER2 and prHER3 gene reporters as seen in Figure 4 was disrupted following depletion of NRF2 in both PEO1 and SKOV3 cell lines to significant levels (Figures 7(c) and 7(d)). These important findings confirmed that tBHQ mediated protein and transcriptional induction of HER receptors was dependent on NRF2 and not by any off NRF2 target effect of tBHQ treatment. Finally, we repeated knockdown of NRF2 either alone or with parallel knockdown of KEAP1 and exposed such cells to targeted immunotherapeutics for 24 and 48 h (Figure 7(e)). We found significant increase in cell death in NRF2 knockdown cells upon exposure to the immunotherapeutics and significant reversal of this response with parallel KEAP1 knockdown.

These results confirmed the transcriptional regulatory role of NRF2 for HER2 and HER3 receptors and illustrated alteration of protein abundance as a result of such transcriptional regulation. These data also confirmed the role of NRF2 in determining overall treatment responses to HER2 targeting immunotherapeutics and hence defining the balance between resistance and sensitivity.

## 4. Discussion

The receptor tyrosine kinases (RTKs), exemplified by HER2/HER3 family receptors, are key regulators of cellular proliferation, differentiation, and survival, as well as determinants of cancer initiation, maintenance, and progression [1–4]. Complexity in understanding the HER2/HER3 activation and signaling arises from the intricate and complex regulation of coexpression of HER2/HER3 receptors and their ligands and the broad spectrum of tumour biochemistry, heterogeneity, and range of sensitivities and resistance exhibited to drugs targeting the HER receptor system [13, 14, 17, 21]. Furthermore, clinical data on HER2/HER3 coexpression profile correlates to some degree with disease-free survival, not only regarding anti-RTK treatment outcome [66], but also by other therapeutic agents [43, 67, 68]. However, it has been suggested that sustained and complete inhibition of HER3 and its output to PI3K/Akt is required for the maximal antitumour effect of HER2 inhibitors [13, 16] and that inhibition of HER2 receptor alone might not generate sufficient anticancer response [16]. Lately, data have accrued to evidence and implicate NRF2 and ROS, in addition to HER2/HER3, in the promotion of cellular proliferation and therapeutic resistance in cancer cells [31, 69, 70]. It is also known that ROS can trigger both the AR and the HER family receptor pathways with concomitant transcriptional upregulation of HER2/HER3 and NRF2 and subsequent elevation and activation of their functions [31, 68–70]. Thus, the HER2/HER3 family receptor signaling pathway is upstream of PI3K/AKT/mTOR pathway [13–16] and has likewise been shown to be upstream of the NRF-AR pathway as well [46, 47, 59, 60, 71, 72]. These highlight the possibility of a more direct, rather than indirect, contact and cross relationship between the HER2/HER3 and NRF2-AR pathways. This crosstalk could be likely and even necessary as RTK dependent growth and metabolism creates ROS, which would require parallel NRF2 dependent antioxidant pathway for its neutralisation. Likewise, the implication of NRF2 in proliferative and cytoprotective pathways may involve RTK dependent signaling [73]. RTK-targeted cancer therapies are compromised or limited when tumour cells circumvent the action of a single agent, and multiple agents due to the readjustments in coexpression of HER2/HER3 receptors, their ligand binding dynamics, or changing preference for the dimerizing partner [17, 21, 61, 74, 75] suggest that the anticancer effect of these agents might be further optimized or be better predicted by effectively limiting HER2/HER3 expression at the DNA level or at least identifying a common regulatory centre of HER2 and HER3 transcription. Thus the identification of factors that mediate or modulate the transcriptional expression of HER2/HER3 will be paramount.

NRF2 has already been implicated in numerous reports as a key contributor to resistance towards anticancer drugs. However, most of these past studies have explored the role of NRF2 in resistance against DNA damaging agents [50, 51, 55]. The present study demonstrates that NRF2 may regulate cancer cell proliferation, susceptibility, and resistance to targeted therapy via transcriptional regulation of* HER2*/*HER3*. To demonstrate the role of NRF2 in RTK signaling and thus in determining responses to targeted therapies, we used HER2 overexpressing (SKOV3) and low expressing (PEO1) ovarian cancer cell lines [57] grown in HER receptor ligand Heregulin and employed pharmacological and genetic activation or inhibition of both NRF2-AR and HER2/HER3 signaling pathways.

Firstly, pharmacological activation of NRF2 with tBHQ enhanced ovarian cancer cell growth and protected cells from cytotoxicity caused by combined HER2-targeted immunotherapeutic agents, Pertuzumab and Trastuzumab. This was also concomitant with the induction of HER2, HER3, and pAKT proteins in oscillatory and dose-dependent fashions, which is consistent with current emerging concepts of transcriptional control and gene expression [70, 76–80]. Furthermore, NRF2 activation-dependent induction of the receptors and their signaling pathway was governed and executed by NRF2 at the transcriptional level of* HER2* and* HER3* genes. Our results from both immunocytochemistry and gene reporter assays of HER2 and HER3 expressions were further supportive and reminiscent of recent reports that revealed that induction of HER2 might repress HER3 expression while its inhibition led to transcriptional induction of HER3 ([13, 14, 63], also see Figure 3(b)). It is clear that tBHQ treatment led to induction of NRF2, its associated antioxidant transcriptional program, and transcriptional and signaling activation of HER2 and HER3 and that this tBHQ response was evidently dependent on NRF2. Thus, NRF2 activation by tBHQ desensitised RTK signaling pathway to inhibitory action of the HER2 targeting immunotherapeutic agents Pertuzumab and Trastuzumab.

**Figure 8:**
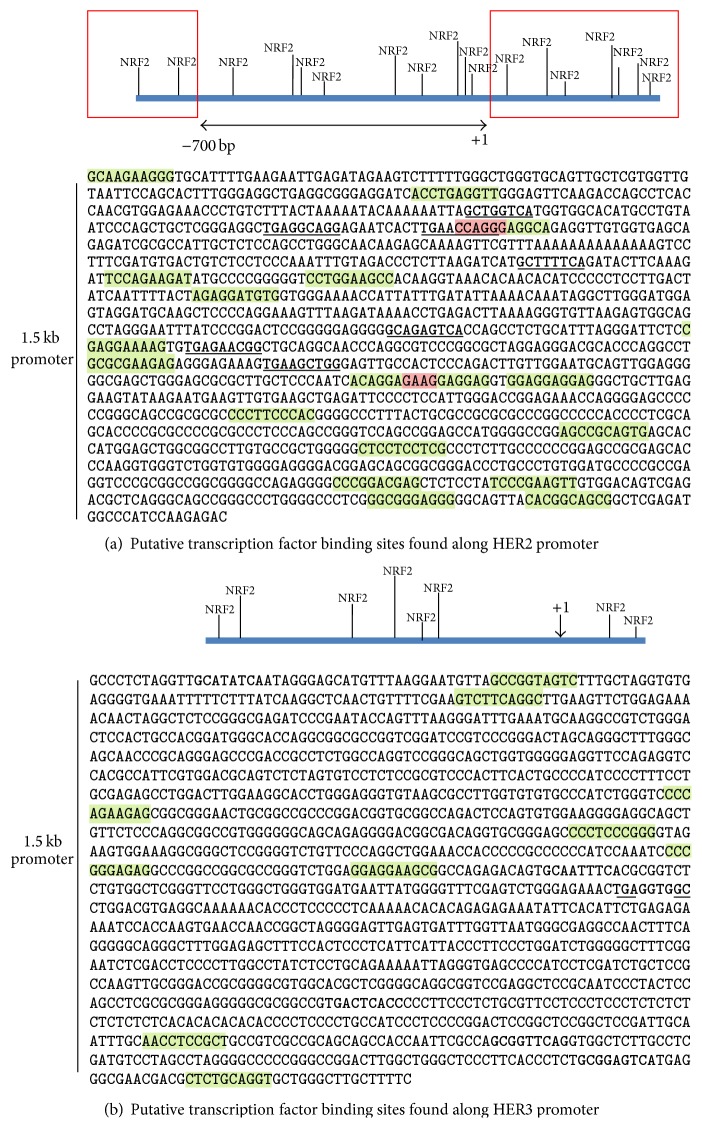
*In silico* analysis of HER2 and HER3 promoter sequences. (a) 1.5 kb promoter region of* HER2* gene was fetched from database (ensemble.org) and subjected to transcriptional factor binding prediction program (http://consite.genereg.net/) to predict for putative NRF2 binding sites as indicated. Line with arrowheads shows the 0.7 kb sequence of HER2 promoter [8], while regions enclosed in rectangles show additional sequences included and cloned in the PGL3 luciferase reporter vector (Promega) because of carrying additional NRF2 binding sites. (b) The same analyses were performed for HER3 promoter. In (a) and (b), +1 indicate the transcriptional start site, sequences highlighted in green show NRF2 binding sites as predicted by ConSite, and sequences in bold represent manual identification of putative NRF2 binding sites based on ARE consensus sequence [9] while those highlighted in pink show overlapping NRF2 binding sites by the two methods mentioned above.

Next, to further investigate and confirm the involvement of NRF2 in the elevation of HER2 and HER3, we took a genetic approach to deplete NRF2 status and function using SiRNA. This approach increased cellular ROS, repressed pNRF2 and HO-1 levels, and even disrupted the tBHQ dependent induction of our ARE reporter system (Figures 5 and 6). In addition, NRF2 depletion by SiRNA caused transcriptional repression of* HER2* and* HER3* leading to lowered expression of HER2, HER3, and pAKT proteins. As an alternative approach, we also cloned and overexpressed individually both NRF2 and KEAP1 genes in our cancer cell lines and found these in either cytoprotection or sensitisation to targeted therapies, respectively (data not shown). Moreover, we illustrated that while knockdown of NRF2 significantly sensitised ovarian cancer cells to targeted immunotherapy, parallel knockdown of KEAP1 reversed this sensitisation. These results support and confirmed our earlier inferred regulatory role of NRF2 in the transcription of* HER2* and* HER3* receptors and its association with alteration of HER2 and HER3 proteins abundance. A recent study has suggested a similar role for NRF2 in regulating the expression of HER2 [73] but fell short of evidencing direct transcriptional regulation as shown in this study. To demonstrate transcriptional modulation of these receptors, we generated and utilised luciferase reporter assays of their proximal promoter sequencing spanning 1.5 kb regions. We performed* in silico* analysis of these upstream regulatory regions for the presence of NRF2 binding and ARE like consensus sequences and found a number of such binding sites (Figure 8). Moreover, a direct interaction of NRF2 and HER2 in regulating the expression of NRF2 target genes, including* HO-1*, via binding of the complex to the ARE of the target genes has been reported [45] which adds credence to our observed downregulation of HER2, HER3, and pAKT as well as HO-1 and pNRF2 levels following our SiRNA-mediated depletion of NRF2. However, further experiments are necessary to confirm the role of NRF2 as a transcription factor for HER receptors.

Thus we have shown that NRF2 regulates HER2 and HER3 signaling pathway to modulate sensitivity to targeted therapies. This demonstrates that NRF2 activation is not only implicated in resistance to genotoxic agents as previously shown [55] but can also lead to resistance to immunotherapies involving Pertuzumab and Trastuzumab, whose actions are very specific to HER2 receptors and unrelated to antioxidant pathway until this study.

## 5. Conclusion

The effectiveness of current anticancer therapies that involve DNA damaging and ROS producing agents is limited, because of NRF2 dependent emergence of cellular resistance to genotoxic agents. On the other hand, targeted anticancer therapeutic agents, while being initially found to be promising, have their own limitations. These include predicting their action and outcome owing to their tight dependence on properties such as cell surface expression of receptors, their dimerizing preferences, presence of ligands, and dynamics of recycling/degradation. This study has found a novel node of regulation between the AR and RTK signaling pathway. As such, the central regulatory node that converges at transcription factor NRF2 presents itself as a very attractive drug target especially in both scenarios of resistance described above. We have presented evidence at the gene expression, protein induction, localisation, and cytotoxicity levels that the two pathways are coregulated and together predict and inform outcomes to targeted immunotherapies and that such responses could be controlled by modulating NRF2 function.

## Figures and Tables

**Table 1:** Antibodies used in the study.

Antibody	Host	Catalogue number	Company
NRF2	Rabbit	Sc-722	Santa Cruz
Phospho-NRF2 S-15	Rabbit	ab76026	Abcam
HER2	Rabbit	2165S	Cell Signalling
HER3	Rabbit	4754S	Cell Signalling
Phospho-HER2 T877	Rabbit	2241S	Cell Signalling
Phospho-AKT 473	Rabbit	4060S	Cell Signalling
BID	Rabbit	2002	Cell Signalling
Phospho-ERK p44/p22	Rabbit	4379	Cell Signalling
Heme oxygenase-1 (HO-1)	Rabbit	Sc-10789	Santa Cruz
Alexa Fluor 488 conjugated secondary antibody	Rabbit	ab150077	Abcam
Alexa Fluor 568 conjugated secondary antibody	Rabbit	ab175471	Abcam
